# The Diagnostic Performance of Machine Learning-Based Radiomics of DCE-MRI in Predicting Axillary Lymph Node Metastasis in Breast Cancer: A Meta-Analysis

**DOI:** 10.3389/fonc.2022.799209

**Published:** 2022-02-04

**Authors:** Jing Zhang, Longchao Li, Xia Zhe, Min Tang, Xiaoling Zhang, Xiaoyan Lei, Li Zhang

**Affiliations:** Department of MRI, Shaanxi Provincial People’s Hospital, Xi’an, China

**Keywords:** breast cancer, axillary lymph node metastasis, radiomics, machine learning, dynamic contrast-enhanced magnetic resonance imaging, meta-analysis

## Abstract

**Objective:**

The aim of this study was to perform a meta‐analysis to evaluate the diagnostic performance of machine learning(ML)-based radiomics of dynamic contrast-enhanced (DCE) magnetic resonance imaging (MRI) DCE-MRI in predicting axillary lymph node metastasis (ALNM) and sentinel lymph node metastasis(SLNM) in breast cancer.

**Methods:**

English and Chinese databases were searched for original studies. The Quality Assessment of Diagnostic Accuracy Studies (QUADAS-2) and Radiomics Quality Score (RQS) were used to assess the methodological quality of the included studies. The pooled sensitivity, specificity, diagnostic odds ratio (DOR), and area under the curve (AUC) were used to summarize the diagnostic accuracy. Spearman’s correlation coefficient and subgroup analysis were performed to investigate the cause of the heterogeneity.

**Results:**

Thirteen studies (1618 participants) were included in this meta-analysis. The pooled sensitivity, specificity, DOR, and AUC with 95% confidence intervals were 0.82 (0.75, 0.87), 0.83 (0.74, 0.89), 21.56 (10.60, 43.85), and 0.89 (0.86, 0.91), respectively. The meta-analysis showed significant heterogeneity among the included studies. There was no threshold effect in the test. The result of subgroup analysis showed that ML, 3.0 T, area of interest comprising the ALN, being manually drawn, and including ALNs and combined sentinel lymph node (SLN)s and ALNs groups could slightly improve diagnostic performance compared to deep learning, 1.5 T, area of interest comprising the breast tumor, semiautomatic scanning, and the SLN, respectively.

**Conclusions:**

ML-based radiomics of DCE-MRI has the potential to predict ALNM and SLNM accurately. The heterogeneity of the ALNM and SLNM diagnoses included between the studies is a major limitation.

## Highlights

Overall pooled AUC was 0.89 with (95%CI: 0.86, 0.91)

ML, 3.0 T, being manually drawn, using biopsy as gold standard could improve diagnostic performance compared to deep learning, 1.5 T, semiautomatic scanning, pathology, respectively.

## Introduction

*Axillary lymph node metastasis (ALNM) is common in breast cancer patients and determines the clinical stage, treatment plans, surgical procedure and patient outcome (1, 2). Currently, the axillary lymph node (ALN) status of patients with breast cancer is diagnosed by sentinel lymph node biopsy (SLNB) and axillary lymph node dissection (ALND). However, these procedures are not risk-free operations and can potentially lead to implantation metastasis (3). Therefore, it is essential to explore a noninvasive approach for assessing ALNM to reduce the incidence of surgical complications and improve the patient’s quality of life.

Dynamic contrast-enhanced (DCE) magnetic resonance imaging (MRI) has generally been well accepted and routinely used for breast cancer staging (4, 5). For predicting ALNM, previous studies of DCE-MRI have primarily focused on node size, cortical thickness, disappearance of lymph parenchyma, and enhancement patterns (6). Unfortunately, early diagnosis of ALNM through DCE-MRI is not yet ideal since it is limited by subjective factors, such as the radiologist’s experience and knowledge level. Additionally, subtle changes, such as cell density, morphology, and microtissue structure, in ALNM might not be apparent to the naked eye (7, 8).

In recent years, radiomics and machine learning (ML) models have become increasingly popular for analyzing diagnostic images (9, 10). The ability of radiomics analysis to maximize the number of features in quantitative images has excellent potential for evaluating ALNM in breast cancer patients (11–15).

However, because of the small sample sizes of previous studies, statistical research has been limited, and research results have also varied from study to study. Thus, it is necessary to perform a meta‐analysis to further evaluate the diagnostic performance of ML-based radiomics of DCE-MRI in predicting ALNM and SLNM in breast cancer.

## Materials and Methods

We conducted and reported this meta-analysis based on the PRISMA (Preferred Reporting Items for Systematic Reviews and Meta-Analyses) guidelines (16).

### Literature Search

The PubMed, Embase, Web of Science, and Cochrane Library databases and four Chinese databases [VIP, CNKI, Wanfang and Chinese BioMedical Literature Databases (CBM)] were searched by two observers independently to identify studies. The search was performed on June 23, 2021, without a start date limit. The study search was conducted using the following keywords: “magnetic resonance imaging”, “MRI”, “MRI scans”, “breast cancer”, “breast carcinoma”, “metastasis”, “machine learning”, “radiomics” and “lymph node”. MeSH terms and variations of each term were used. Moreover, we restricted the studies to those published in English or Chinese and performed a manual search of the related articles’ reference lists to identify other articles that might meet the inclusion criteria. Endnote software, version X9, was used to manage all records. Disagreements were discussed and resolved to reach a consensus.

### Study Selection

The titles and abstracts of potentially relevant studies were screened for appropriateness by two reviewers(Z-J and Z-L). Inconsistencies were discussed by the reviewers, and consensus was reached.

All of the studies were selected according to the following criteria: (a) original research studies; (b) patients with breast cancer were enrolled who were confirmed to have ALNM or SLNM by biopsy or histopathology; (c) ML-based DCE-MRI applied to classify ALNM or SLNM using radiomics; and (d) data are sufficient to reconstruct the 2×2 contingency table to estimate the sensitivity and specificity of the diagnosis.

Studies were excluded if: (a) reviews, editorials, abstracts, animal studies, and conference presentations; and (b) multiple reports published for the same population (in this case, the publication with the most details was chosen to be included in this meta-analysis).

### Data Extraction

Relevant data were extracted from each study, including the first author, publication year, sample size, magnetic field strength, information about radiomics and ML pipeline, data sources and reference standards, detailed information on lesion segmentation, contrast agents, and DCE phases. For each study, the true positive (TP), false-positive (FP), false negative (FN), and true negative (TN) values were extracted, and a pairwise (2×2) contingency table was created.

### Data Quality Assessment

The Quality Assessment of Diagnostic Accuracy Studies (QUADAS-2) and Radiomics Quality Score (RQS) were used to assess the methodological quality of the included studies and the risk of bias at the study level, respectively (17, 18). RQS items comprise: (a) image acquisition; (b) radiomics feature extraction; (c) data modeling; (d) model validation; and (e) data sharing. Each of the 16 items (**Table 1**) of the RQS is rated, resulting in a total of points ranging from −8 to 36, with −8 defined as 0% and 36 defined as 100% (18).

**Table 1:** Elements of the RQS and average rating achieved by the studies included in this meta-analysis.

RQS scoring item	Interpretation	Average
Image Protocol	+1 for well documented protocols, +1 for publicly available protocols	0.92
Multiple Segmentations	+1 if segmented multiple times (different physicians, algorithms, or perturbation of regions of interest)	0.62
Phantom Study	+1 if texture phantoms were used for feature robustness assessment	0.62
Multiple Time Points	+1 multiple time points for feature robustness assessment	0.08
Feature Reduction	−3 if nothing, +3 if either feature reduction or correction for multiple testing	3
Non Radiomics	+1 if multivariable analysis with non-radiomics features	0.54
Biological Correlates	+1 if present	0.08
Cut-off	+1 if cutoff either pre-defined or at median or continuous risk variable reported	0.15
Discrimination and Resampling	+1 for discrimination statistic and statistical significance, +1 if resampling applied	0.15
Calibration	+1 for calibration statistic and statistical significance, +1 if resampling applied	0.08
Prospective	+7 for prospective validation within a registered study	0
Validation	−5 if no validation/+2 for internal validation/+3 for external validation/+4 two external validation	0.38
	datasets or validation of previously published signature/+5 validation on ≥3 datasets from >1 institute	
Gold Standard	+2 for comparison to gold standard	2
Clinical Utility	+2 for reporting potential clinical utility	1.69
Cost-effectiveness	+1 for cost-effectiveness analysis	0.08
Open Science	+1 for open-source scans, +1 for open-source segmentations, +1 for open-source code, +1 open-source representative segmentations and features	1

The QUADAS-2 tool consists of: (a) patient selection; (b) index test; (c) reference standard; and (d) flow and timing. Two independent reviewers (L-LC and Z-L) conducted the quality assessment, and disagreements were discussed with a third reviewer (T-M) to reach a consensus.

### Statistical Analysis

This meta-analysis was conducted *via* Stata software, version 16.0, Review Manager software, version 5.3, and the Open Meta-analyst software tool. The predictive accuracy was quantified using pooled sensitivity, specificity, diagnostic odds ratio (DOR), positive likelihood ratio (PLR) and negative likelihood ratio (NLR) with 95% confidence intervals (CIs). The summary receiver operating characteristic curve (SROC) and area under the curve (AUC) were used to summarize the diagnostic accuracy.

Q and I^2^ were calculated to estimate the heterogeneity among the studies included in this meta-analysis. I^2^ values of 0 to 25%, 25 to 50%, 50 to 75% and >75% represent very low, low, medium and high heterogeneity, respectively. Pooling studies and effect size were evaluated using a random-effects model, indicating that estimating the distribution of true effects between studies considers heterogeneity (19). If there was obvious heterogeneity, Spearman’s correlation coefficient was used to assess the threshold effect between the sensitivity logit and the specificity logit. Subgroup analysis was performed to further investigate the cause of the heterogeneity. The following covariates were used to explain factors that could contribute to heterogeneity: (a) 1.5 T MR vs. 3.0 T MR; (b) Pathology of SLNB or ALND vs. Pathology of ALND; (c) deep learning vs. ML; (d) ALN vs. SLN vs. ALN and SLN; (e) area of interest (ROI) including ALN vs. ROI including breast cancer; and (f) semiautomatic vs. manual drawing; (g) support vector machines(SVM) vs. logistic regression(LR); (h) Siemens MR equipment vs. GE MR equipment.

In addition, the sensitivity analysis was assessed by eliminating the included studies one by one. The effective sample size funnel plot described by Deek’s test was used to estimate publication bias (20).

### Clinical Utility

A Fagan plot was used to assess the clinical utility, which provided the posttest probability (P post) of ALNM when pretest probabilities (P pre, suspicion of ALNM) were calculated (21).

## Results

### Literature Search

**Figure 1:**
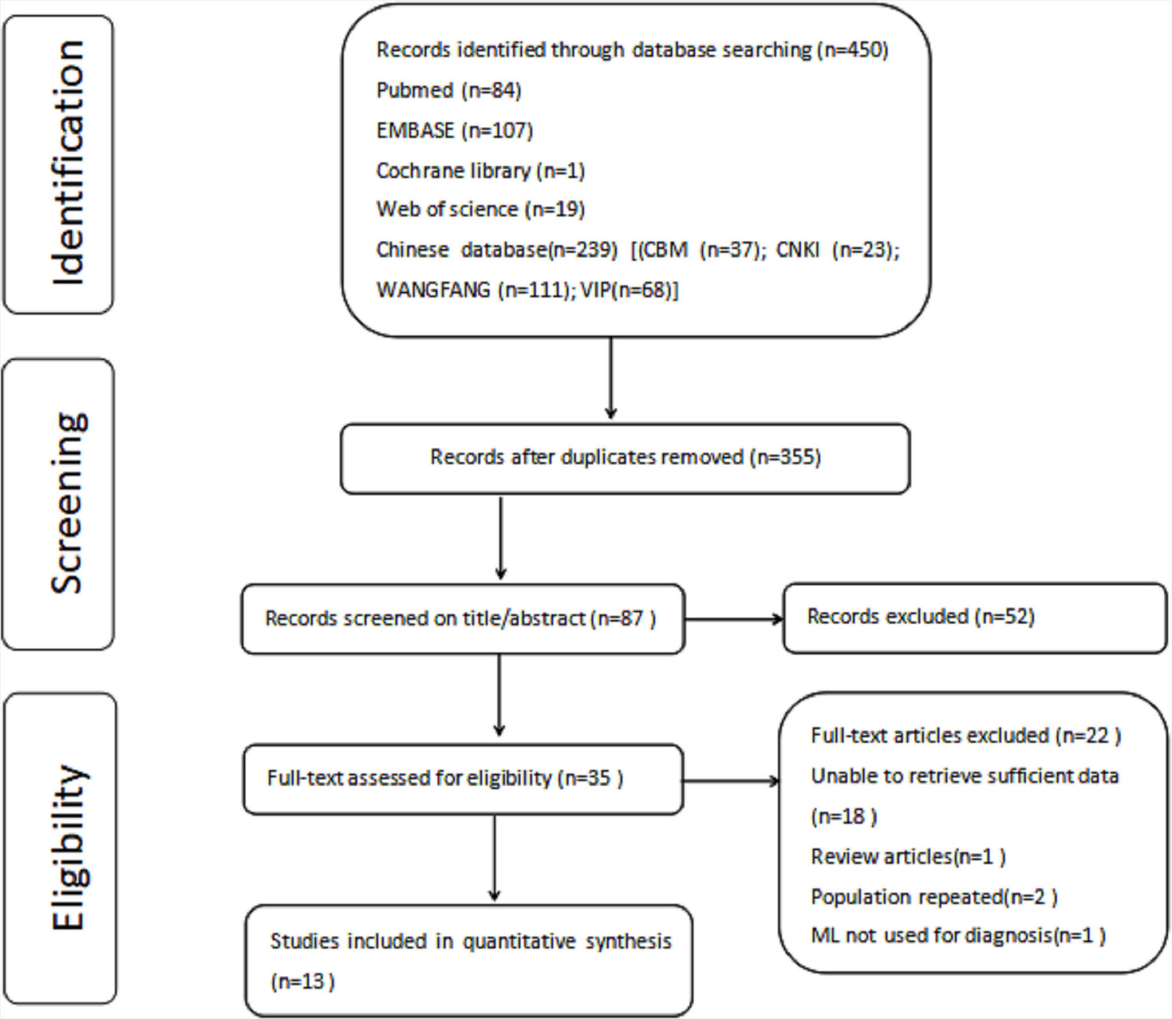
Flow diagram of study selection for meta-analysis.

The complete literature search flowchart is presented in **Figure 1**.

According to the search strategy described above, 450 potentially eligible citations were identified. After removing 95 duplicate records, 355 titles were considered. After the title and abstract evaluation, 268 citations were omitted because they did not meet the inclusion criteria. After revision, 22 articles were excluded, leaving 13 articles for inclusion in the meta-analysis (11–15, 22–29).

### Data Quality Assessment

**Figure 2:**
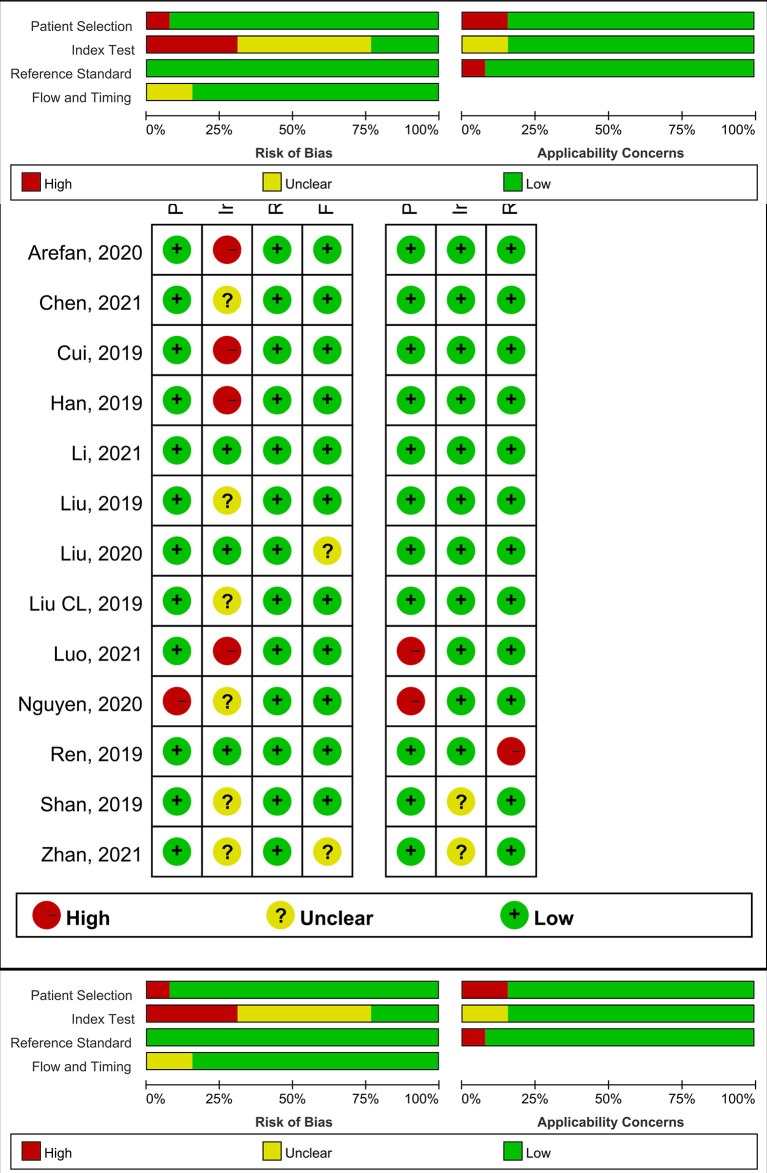
The risk of bias (left) and concerns for applicability (right) for each included study using QUADAS-2.

The 13 studies achieved an average RQS range of 11.38, a median of 13, and a range of 5 to 15. The mean RQS proportion was 13.9%, with a maximum of 41.7%. **Table 1** summarizes the mean scores for each dimension, and **Table S1 (Supplement Materials)** shows the RQS for each study and the individual scores for each study. None of the included articles employed prospective validation, and only one study evaluated the cost-effectiveness of radiomics (25). No studies publicly shared segmentation, functionality, or code. Generally, the data quality was considered acceptable, and the details of the risk of bias and applicability concerns of the included studies are presented in **Figure 2**.

### Characteristics of the Included Studies

The characteristics of the included studies are summarized in **Tables 2** and **3**. Detailed two-by-two contingency tables of every study are shown in **Table S2 (Supplement Materials)**. The 13 studies included in this meta-analysis had 2253 patients, and 1618 participants in valid or test set. All of the studies used retrospectively collected data. The models in these studies included ML (n=11) and deep learning (n=2) for unsupervised learning. Of these models, the ML algorithm comprised different types of logistic regression models (12, 15, 22, 23, 28), convolutional neural network models (26, 27), multiple classifier systems (11, 13, 14, 24, 25), and support vector machine models (29).

**Table 2:** Baseline characteristic of included studies (1).

Study	NO.patient		Magnetic field	Contrast agent	Phase	Data source
Arefan, 2020 (11)	154	Siemens	3.0T	Magnevist	CE2	Single institution
Chen, 2021 (12)	140	GE	3.0T	GD-DTPA	the strongest enhanced phase	Single institution
Cui, 2019 (13)	115	Siemens	3.0T	GD-DTPA	CE2	Single institution
Han, 2019 (14)	411	GE	1.5T	Omniscan	CE1	Single institution
Liu CL, 2019 (23)	163	GE	1.5T	Magnevist	One precontrast and four post-contrast phases	Single institution
Liu, 2019 (24)	62	GE	3.0T	GD-DTPA	the strongest enhanced phase	Single institution
Liu, 2020 (22)	164	GE	3.0T	GD-DTPA	the strongest enhanced phase	Single institution
Nguyen, 2020 (26)	357	GE	1.5T	gadopentetate dimeglumine /Gadavist	a single precontrast and four serial dynamic image	Two institution
Zhan, 2021 (29)	166	Siemens	3.0T	Omniscan	the strongest enhanced phase	Single institution
Shan,2019 (28)	196	Siemens	3.0T	Gd‐DTPA	CE2	Single institution
Luo,2021 (25)	67	Siemens	3.0T	Gadolinium Diamine and Cardiamine Sodium	CE1	Single institution
Ren, 2020 (27)	61	GE	1.5T	Gadavist,	CE1	Single institution
Li, 2021 (15)	197	Philips	1.5T	Gadoteric acid meglumine salt	the early-and delayed-phase	Single institution

CE1, the first postcontrast images; CE2, the second postcontrast phase.

**Table 3:** Baseline characteristic of included studies (2).

Study	Technique used for feature selection	Classification	Reference standard	Segmentation lesion	Tumor segmentation	Validation
Arefan, 2020 (11)	LASSO	LDA, RF, NB,KNN, SVM	SLNB or ALND	2D, 3D	semi-automatically	Test set, 10-fold cross-validation
Chen, 2021 (12)	LASSO+10fold crossvalidation	LR	Pathology	3D	manually	10-fold cross-validation
Cui, 2019 (13)	LASSO	SVM, KNN, LDA	SLNB or ALND	2D, 3D, 4D	semi-automatically	cross-fold validation
Han, 2019 (14)	LASSO+LOOCV	SVM	Pathology	3D	manually	6-fold validation
Liu CL, 2019 (23)	LASSO+3fold crossvalidation	LR	Pathology	3D	manually	10-fold cross-validation
Liu, 2019 (24)	The select K best+LASSO	SVM, Xgboost, LR	Pathology	3D	manually	cross-fold validation
Liu, 2020 (22)	LASS0	LR	Pathology	3D	manually	NOT REPORTED
Nguyen, 2020 (26)	CNN		Pathology	3D	semi-automatically	10-fold cross-validation, Test set
Zhan, 2021 (29)	Spearman correlation analysis	SVM-RF	SLNB or ALND	3D	manually	5-fold validation
Shan,2019 (28)	One-way analysis of variance+Wilcoxon rank sum test+correlation test+LASSO	LR	SLNB or ALND	3D	manually	Confusion matrix
Luo,2021 (25)	LASSO	linear discriminant analysis and leave-one-case-out-cross-validation	Pathology	3D	manually	10-fold cross-validation
Ren, 2020 (27)	CNN		PET/CT	2D	semi-automatically	5-fold cross-validation
Li, 2021 (15)	Spearman+LASSO	LR	SLNB or ALND or Pathlogy	3D	manually	5-fold cross-validation

LR, logistic regression; CNN, convolutional neural network; SVM, support vector machine; LDA, linear dis-criminant analysis; RF, random forest; NB, naive Bayes; KNN, K-nearest neighbor; LASSO, least absolute shrinkage and selection operator.

In 13 studies, different phase analysis methods of DCE were used, including the strongest enhanced phase, the second postcontrast phase, the first postcontrast images, and two-phase images in 4 (12, 22, 23, 29), 3 (11, 13, 28), 3 (14, 25, 27), and 3 (15, 24, 26) studies, respectively. 3 T scanners were used in 8 studies (11–13, 22, 24, 25, 28, 29), and 1.5 T MR was used in 5 studies (14, 15, 23, 26, 27). Seven studies (13–15, 23, 24, 28, 29) employed SLNB or ALND to serve as the reference standard, while the remaining 3 studies (12, 25, 26) were based on ALND. Additionly, PET/CT (27) and ultrasound-guided fine-needle aspiration or ALND (11) of 1 study, respectively. Five studies (11, 12, 25–27) focused specifically on ALN, whereas 3 studies (22–24) focused on SLN, the remaining 5 studies (13–15, 28, 29) focused on ALN and SLN. Only 4 studies (11, 13, 26, 27) used semiautomatic segmentation, and 9 studies (12, 14, 15, 22–25, 28, 29) used manual ROIs. The ROIs of the breast tumor area and ALN area were employed in 10 studies (11–15, 22–24, 26, 29) and 3 studies (25, 27, 28), respectively.

### Data Analysis

**Figure 3:**
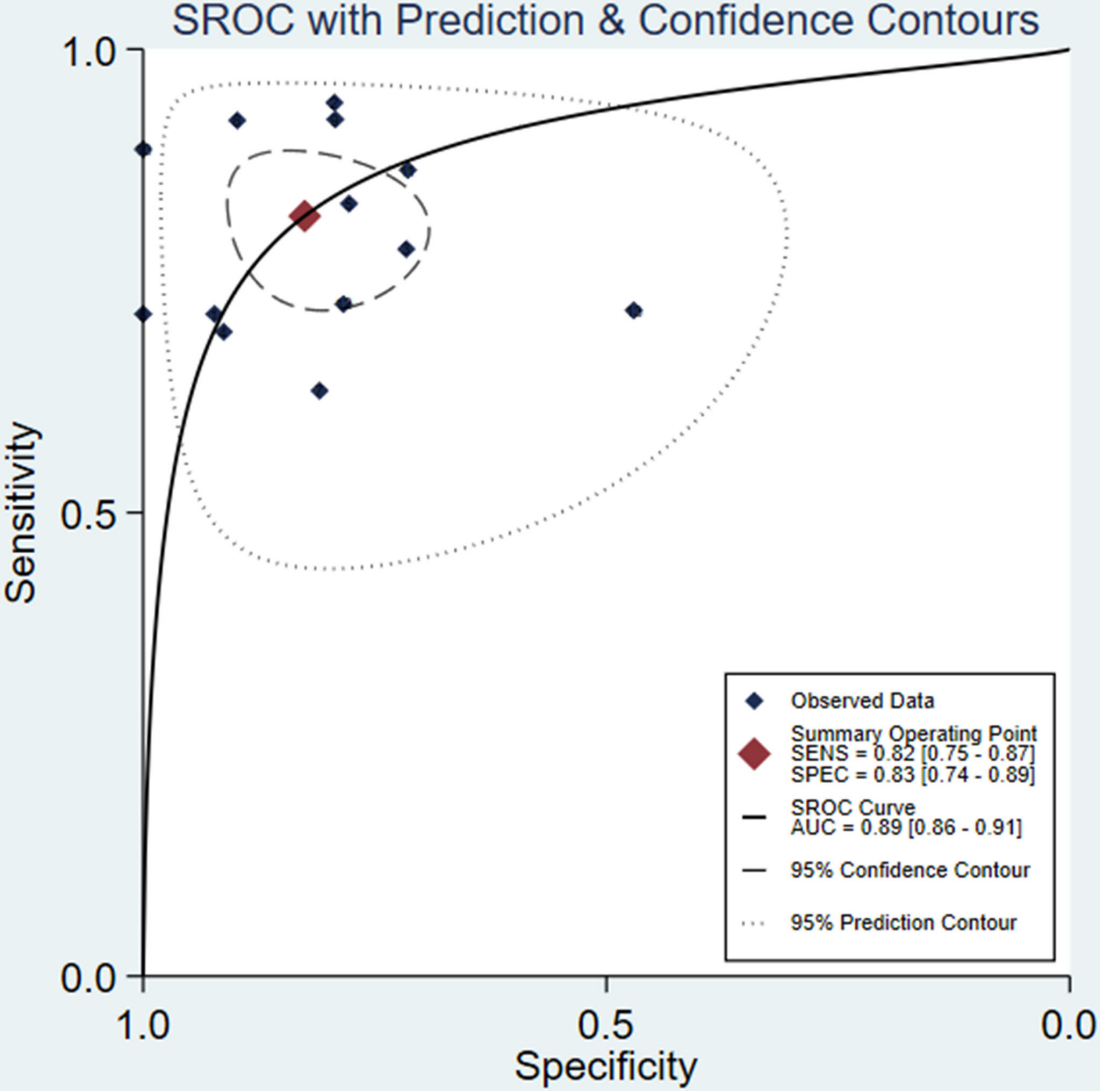
Hierarchical summary receiver operating characteristic (SROC). curve of the diagnostic performance of ML-based radiomics of DCE-MRI in predicting ALNM in breast cancer.

For all 13 studies, the mean values and 95% CIs of pooled sensitivity, specificity, PLR, NLR, and DOR for the radiomics signature based on DCE-MRI in assessing ALNM and SLNM in breast cancer were 0.82 (0.75, 0.87), 0.83 (0.74, 0.89), 4.70 (3.01, 7.35), 0.22 (0.15, 0.31), and 21.56 (10.60, 43.85), respectively (**Table 4**). The ML models for ALNM and SLNM in breast cancer showed an overall pooled AUC=0.89 (0.86, 0.91) (**Figure 3**).

**Table 4:** The results of subgroup analysis.

Analysis	No. of study	Sensitivity	Specificity	PLR	NLR	DOR
Overall	13	0.82 (0.75,0.87)	0.83 (0.74,0.89)	4.70 (3.01,7.35)	0.22 (0.15,0.31)	21.56 (10.60,43.85)
DL vs ML						
ML	11	0.80 (0.73,0.86)	0.83 (0.76,0.88)	4.45 (3.27,6.07)	0.21 (0.14,0.32)	22.82 (12.33,42.23)
DL	2	0.84 (0.53,0.96)	0.65 (0.31,0.89)	2.45 (0.76,7.85)	0.24 (0.04,1.45)	9.95 (0.51,192.87)
Biopsy/vs Pathology						
Biopsy	6	0.85 (0.74,0.92)	0.82 (0.75,0.88)	4.50 (3.29,6.15)	0.17 (0.09,0.31)	29.17 (13.34,63.81)
Pathology	7	0.77 (0.68,0.84)	0.79 (0.62,0.89)	3.63 (1.93,6.83)	0.28 (0.16,0.52)	13.95 (4.17,46.66)
1.5T vs 3.0T						
3.0T	8	0.82 (0.72,0.89)	0.83 (0.76,0.88)	4.62 (3.16,6.75)	0.18 (0.10,0.34)	30.09 (11.87,76.28)
1.5T	5	0.78 (0.69,0.85)	0.76 (0.58,0.88)	3.37 (1.74,6.55)	0.26 (0.11,0.61)	12.71 (3.56,45.41)
SLN vs ALN						
ALN	10	0.82 (0.75,0.87)	0.81 (0.70,0.88)	4.27 (2.60,7.03)	0.20 (0.11,0.38)	23.62 (8.99,62.04)
SLN	3	0.71 (0.56,0.83)	0.80 (0.68,0.88)	3.74 (2.11,6.31)	0.27 (0.16,0.46)	12.17 (4.58,32,36)
Segmentation method						
Semiautomatic	5	0.82 (0.70,0.90)	0.74 (0.56,0.87)	3.26 (1.60,6.61)	0.21 (0.07,0.60)	15.95 (3.63,70.04)
Manually drawing	8	0.80 (0.71,0.86)	0.84 (0.75,0.90)	4.82 (3.08,7.53)	0.23 (0,16,0.33)	23.59 (9.22,47.57)
different ROI						
Lymph	3	0.85 (0.68,0.94)	0.81 (0.71,0.88)	4.30 (2.59,7.15)	0.17 (0.05,0.54)	38.12 (7.06)
Breast Cancer	10	0.79 (0.71,0.85)	0.80 (0.67,0.89)	4.02 (2.38,6.79)	0.23 (0.13,0.42)	17.62 (6.68,46.49)
Different algorithms of ML						
SVM	5	0.81 (0.70,0.89)	0.76 (0.70,0.81)	3.32 (2.64,4.17)	0.20 (0.10,0.39)	15.27 (7.49,31.13)
LR	5	0.75 (0.65,0.82)	0.88 (0.77,0.94)	5.72 (3.13,10.44)	0.29 (0.20,0.43)	22.56 (9.15,55.62)
Different MR equipment						
Siemens	5	0.88 (0.77,0.94)	0.82 (0.73,0.89)	4.74 (2.93,7.66)	0.14 (0.07,0.30)	42.37 (11.97,149.91)
GE	7	0.77 (0.68,0.84)	0.75 (0.61,0.86)	3.21 (1.79,5.75)	0.28 (0.13,0.62)	12.17 (4.03,36.75)

PLR, positive likelihood ratio; NLR, negative likelihood ratio; DOR, diagnostic odds ratio; SVM,support vector machines; LR,logistic regression.

### Exploration of Heterogeneity

**Figure 4:**
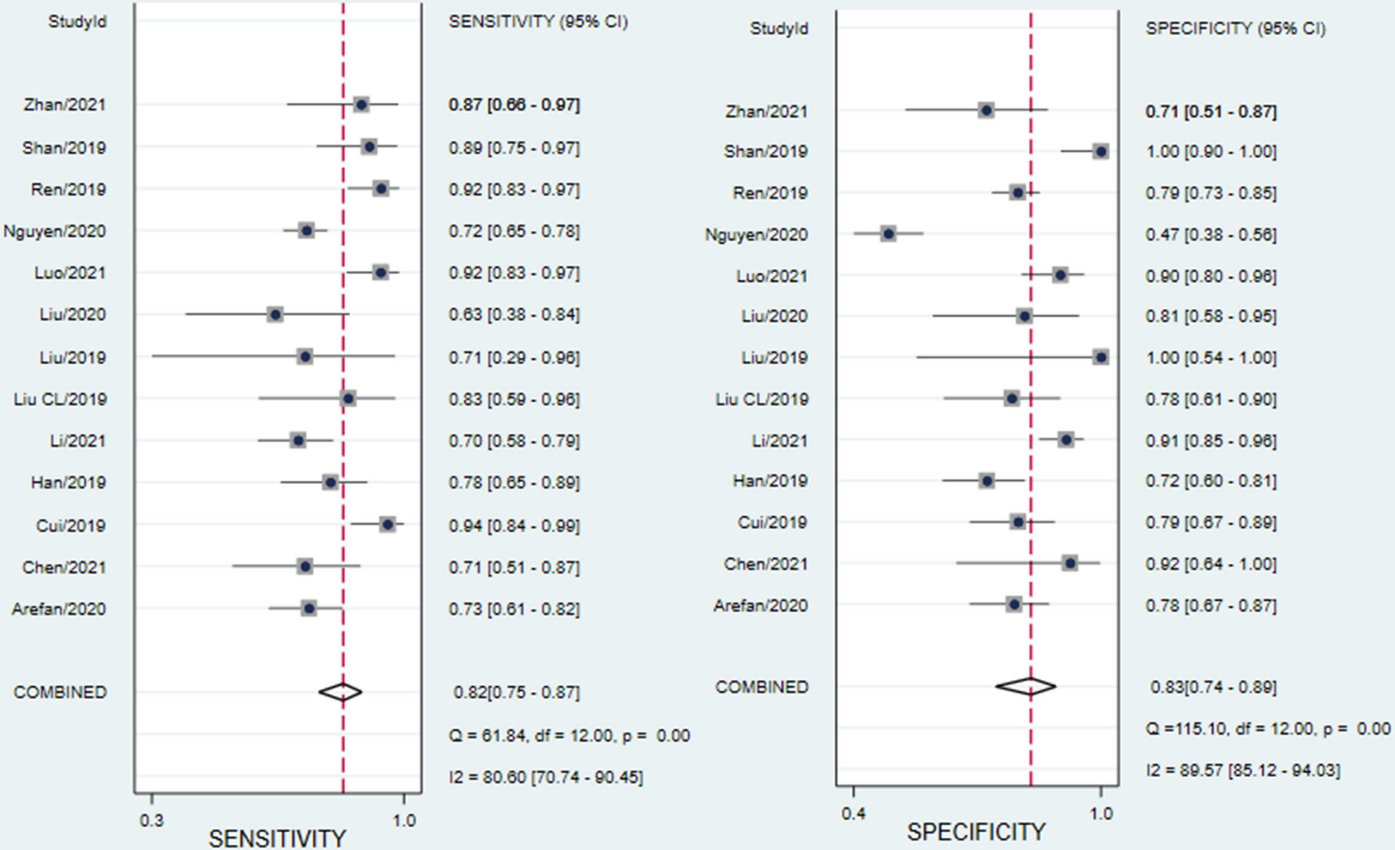
Forest plots of the sensitivity and specificity of ML-based radiomics of DCE-MRI in predicting ALNM in breast cancer. I^2^>50% indicated substantial heterogeneity in the diagnostic parameters across studies.

There was significant heterogeneity in sensitivity (I^2 =^ 80.6%) and specificity (I^2 =^ 89.57%). As shown in **Figure 4**, the results of the diagnostic threshold analysis showed that there is no threshold effect because Spearman’s correlation coefficient was 0.181, and the *P* value was 0.553.

Subgroup analysis was also performed by comparing studies with the different variables. **Table 4** shows the results of the analysis for subgroups.

Studies (n =11) using ML had higher specificity (0.83 vs. 0.65) and an equivalent sensitivity (0.80 vs. 0.84) compared to studies (n=2) that used deep learning. The studies that used a 3.0 T MR had higher sensitivity (0.82 vs. 0.78) and specificity(0.83 vs. 0.76) than those that used 1.5 T MR. Five studies with SLNB or ALND as the gold standard had an equivalent sensitivity (0.82 vs. 0.80) and specificity(0.82 vs. 0.80) with studies(n=3) with ALND as reference standard. Studies (n=3) that only included SLNs had lowest sensitivity (0.71 vs. 0.81 vs.0.84) and an similar specificity (0.80 vs. 0.78 vs. 0.82) in among studies that only included ALNs and combined SLNs and ALNs groups. Eight manually drawn studies had higher specificity (0.84 vs. 0.74) and equivalent sensitivity (0.80 vs. 0.82) than studies (n=5) using semiautomatic segmentation. Studies (n =3) with LN as the ROI had higher sensitivity (0.85 vs. 0.79) and equivalent specificity (0.81 vs. 0.80) compared to studies (n =10) with breast cancer as the ROI. ML including the different algorithms in models, SVM algorithms had higher sensitivity (0.81 vs. 0.75) and lower specificity (0.75 vs. 0.88) compared to studies with LR algorithms. The studies that used Siemens MR equipment had higher sensitivity (0.88 vs. 0.77) and specificity (0.82 vs. 0.75) than studies used GE equipment. The corresponding forest plots are presented in **Figures S1–8 (Supplement Materials)**.

### Sensitivity Analyses

There were no significant changes when eliminating the included studies one by one. The results of sensitivity analyses for each study are shown in **Table S3 (Supplement Materials)**.

### Publication Bias

**Figure 5:**
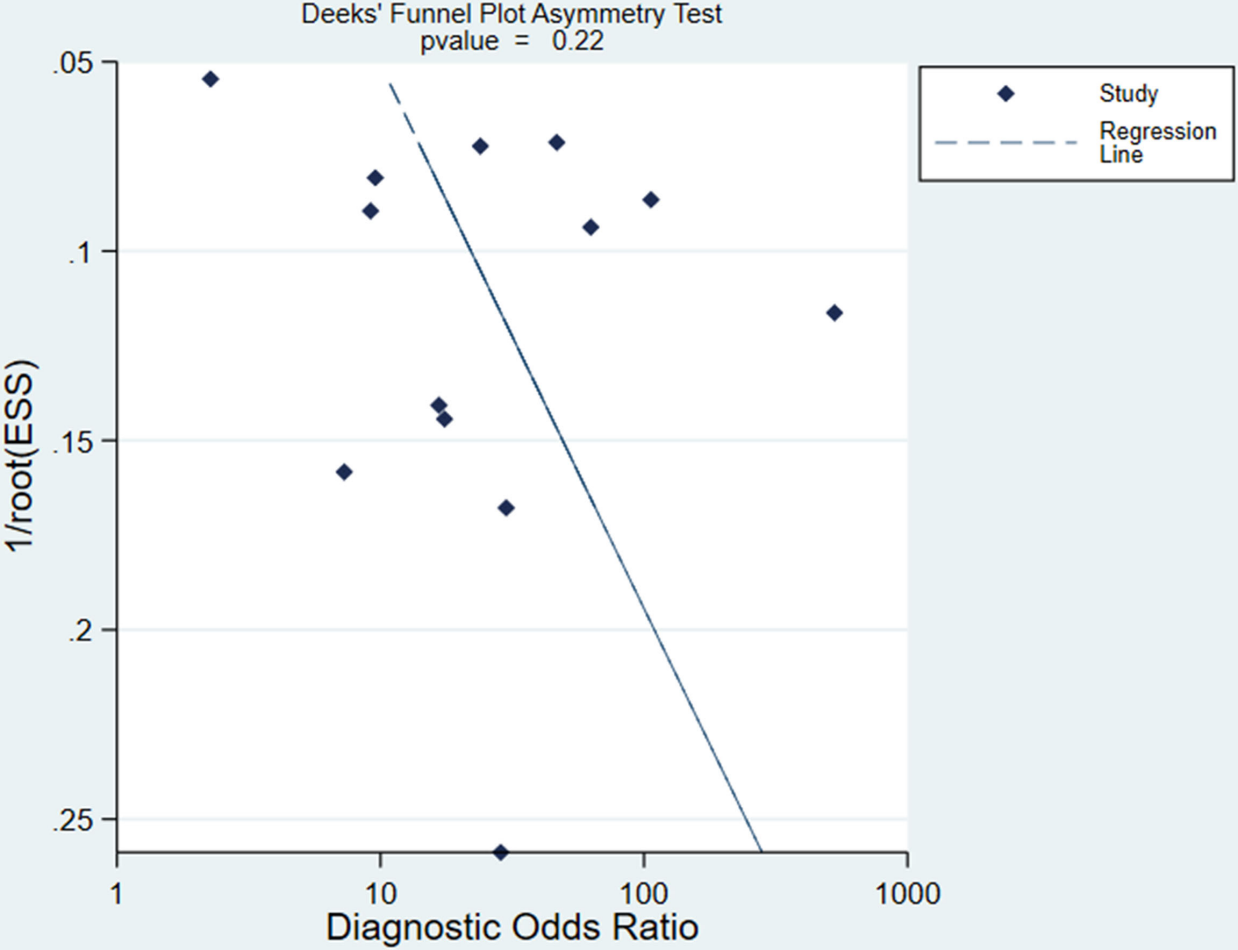
Deeks funnel plot shows the likelihood of publication bias is low with a P value of 0.22. ESS, effective sample size.

There was no publication bias based on the Deeks funnel plot (*P*=0.22) (**Figure 5**) (20).

### Clinical Utility

**Figure 6:**
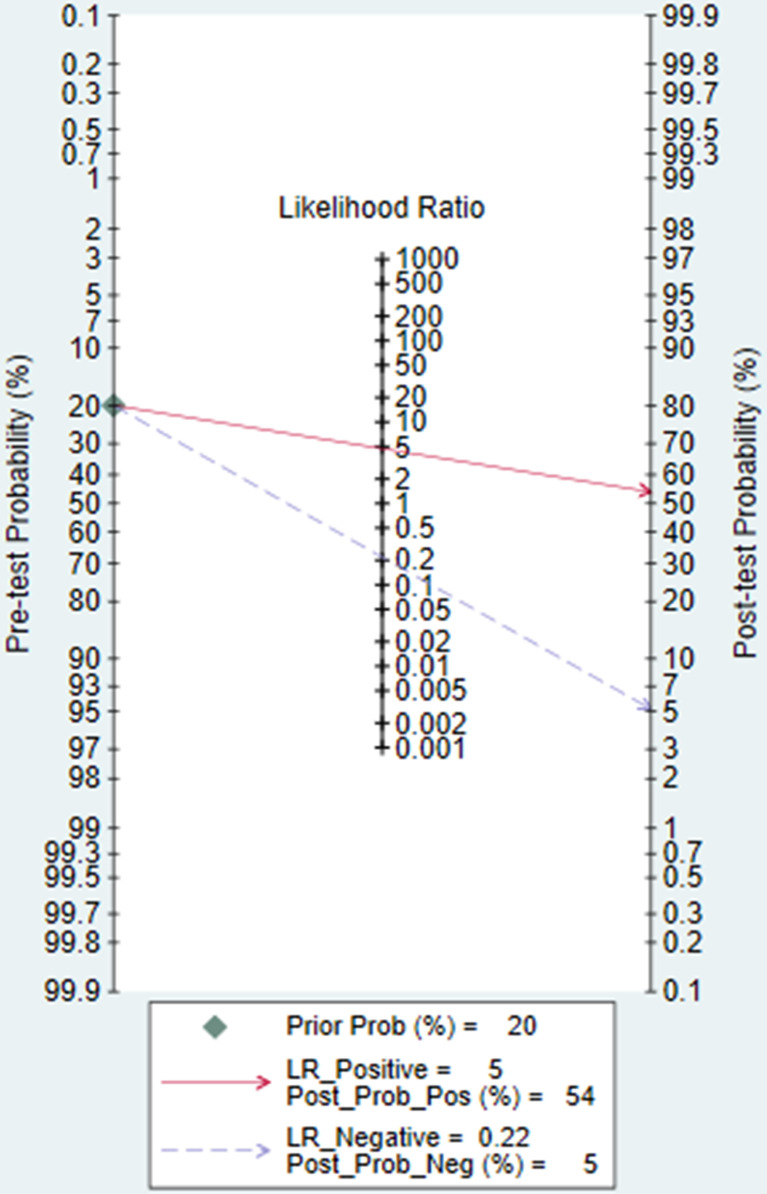
Fagan plot of ML-based radiomics models of DCE-MRI in predicting ALNM in breast cancer.

Using an ML-based radiomics DCE-MRI model would increase the posttest probability to 54 from 20% with a PLR of 5 when the pretest was positive and would reduce the posttest probability to 5% with an NLR of 0.22 when the pretest was negative (**Figure 6**).

## Discussion

In our meta-analysis, radiomics DCE-MRI showed promising results for ALNM characterization, with a pooled sensitivity, specificity, and AUC of 0.82, 0.83, and 0.89, respectively. This finding indicates that this approach could be considered an effective and accurate tool for ALNM and SLNM prediction.

In the present study, we found that there was obvious heterogeneity between the studies. Indeed, heterogeneity can be caused by many factors, e.g., threshold effect, different magnetic fields, segmentation, etc. In this meta-analysis, the threshold effect was not the source of heterogeneity because Spearman’s coefficient was not significant. Therefore, subgroup analysis was used to determine the source of heterogeneity. Our results demonstrated that studies using 3.0 T MR had better diagnostic performance than studies using 1.5 T MR. We are not surprised by this result. Since high magnetic fields can improve image resolution, they can help to improve diagnostic accuracy. Another subgroup analysis result showed that studies employing ML have slightly better value than those employing deep learning. Deep learning has greater potential for very large datasets with thousands or even millions of instances. In this setting, datasets usually consist of hundreds of patients at most, which is better than with deep learning in this case. Similar findings have been previously reported for ML in other applications (9, 10, 30). However, deep learning only included two studies. Future studies employing deep learning are needed to confirm this conclusion.

ROIs including the ALN area have good diagnostic performance compared with ROIs including the breast tumor area. While an ROI of the ALN is useful to evaluate ALN status, it suffers from some limitations, such as the ALN breast surface coil being mainly concentrated in the breast area; nevertheless, some positive lymph nodes might be located at the edge of the coil, and some might not even be in the imaging range (31). Studies have focused on breast tumors themselves, which could help to avoid the above limitations. Studies with SLNB or ALND as the gold standard had an equivalent sensitivity and specificity with ALND group. The reason may be that the patient with negative SLN, SLNB maybe an effective and accuracy approach. The sensitivity of predict SLNM is lower than that to predict ALNM and the two kinds of LNs. Therefore, for SLNM, the diagnostic performance of this imaging tool might not be satisfactory, as concluded in this meta-analysis. Further studies should investigate how to improve the sensitivity of SLNM. Although studies in which ROIs are manually drawn by radiologists might be more prone to error and user variability, the prediction is still good compared with the semiautomatic segmentation method. However, manual segmentation is time consuming, tedious, and prone to error. In the future, it would be ideal to develop a reliable and validated automatic method. Our results showed that LR algorithm had higher DOR than SVM. Generally, LR and SVM algorithms are all suitable for model construction with small sample sizes and binary variables. However, for ML-based DCE-MRI radiomics in predicting ALNM, the LR algorithm is more recommended for use with our meta-analysis result. We also found that studies using Siemens MR equipment had higher diagnostic performance than using GE equipment. It means different MR equipment maybe affect the diagnostic performance. Therefore, prospective studies compared the two MR equipment are necessary to explore the diagnostic performance of ML-based DCE-MRI radiomics in predicting ALNM and SLNM. In addition, different DCE phases and cross-validation of different multiples could lead to unknown biases. Moreover, other unmentioned differences between studies might contribute to the heterogeneity.

A previous meta-analysis (32) including 3 studies of DCE-MRI (n=187) reported that the mean sensitivity and specificity were 0.88 and 0.73, respectively. Another study (6) included 7 studies using DCE-MRI and reported that the median sensitivity was 0.60 (range 0.33.3–0.97) (31). Our findings showed higher sensitivity than studies that included DCE-MRI. Conventional DCE only included morphology and a few quantitative parameters. However, radiomics could provide many new quantitative imaging markers and help to characterize heterogeneous tumor lesions (33). This method could provide more valuable information to help radiologists to improve detection, diagnosis, staging, and prediction power.

### Limitations

All of the methodological issues followed the Cochrane handbook (34), but there are still some limitations that must be discussed. First, a relatively small number of studies met the selection criteria. The second limitation was the significant heterogeneity, which is an issue similar to that in other meta-analyses of diagnostic accuracy using ML based on radiomics (9, 10, 30).

Furthermore, study characteristics, such as different ROIs, DCE phases, and reference standards, could lead to heterogeneity. Therefore, we employed subgroup analysis to reduce heterogeneity.

Third, while there were some uncertainties in the QUADAS-2 assessment, the overall quality of the study was sufficient for analysis. Thus, this uncertain risk might not have had a significant impact on the outcomes.

Fourth, 3 studies(3/13)showed an RQS score<20%. The mean RQS score obtained by analyzing the articles reviewed in this study was 11.1 (30.1%), indicating moderate overall quality. The most important points were the type of study, biological relevance tests and discussion, validation, comparison with the gold standard, potential clinical utility, economic analysis and open scientific data (**Table 1** and **Table S1**). Fifth, in most studies, the lymph nodes assessed by MR have not been specifically associated with histological findings in a node-to-node manner, which is a difficult problem to solve in clinical practice. And it is inevitable that very small lesions may be missed through DCE-MRI. Sixth, some studies used the SLNB as reference standard, which may be caused some false negative rate. Finally, in this meta-analysis, the PLR, NLR and posttest probability were moderate, which would limit the recommendation of their integration into clinical practice.

### Future

To improve the clinical applicability of future studies utilizing ML-based radiomics for ALNM, several factors must be followed.

First, external validation is usually not performed, which should be seen as a major limitation in the field of study. Therefore, it is advisable to verify the accuracy of these models further. When reporting ML-based radiomics, it is crucial to follow quality guidelines that include external validation. Second, future studies should also consider expanding datasets from multiple centers to overcome imbalances caused by oversampling small samples and to improve classifier performance. Third, the variation process might affect bias. There are significant variations in the number of features selected, the risk of overfitting and redundancy, and the preprocessing steps (such as manual segmentation), reducing reproducibility. In addition, the different DCE phases should be considered. Therefore, it is necessary to build better radiomics and ML paper standards to establish image acquisition, segmentation, feature engineering, statistical analysis and report format standardization to achieve reproducibility and facilitate the search for radiomics (35). Finally, the ALNM and SLNM prediction model was constructed with a combination of MR radiomics and DCE quantitative parameter and clinical characteristic data to further explore more precise predictions and to improve the clinical utility for ALNM and SLNM.

## Conclusion

Our results indicated that ML-based DCE-MRI radiomics indicates good diagnostic performance in predicting ALNM and SLNM in breast cancer with high sensitivity and specificity. Nevertheless, due to the heterogeneity of the included studies, caution should be taken when applying the results.

## Data Availability Statement

The original contributions presented in the study are included in the article/**Supplementary Material**. Further inquiries can be directed to the corresponding author.

## Author Contributions

JZ, LL, and LZ conceived and designed the study strategy. LL and MT worked for study search. JZ an LZ worked for study selection. LL and LZ extracted data from each included study and assessed the study quality. XL and JZ prepared the tables and all figures. XLZ and XZ worked as the supervisor and made arbitration for all possible disagreements. All authors have read and approved the content.

## Funding

This work was supported by the Talent Support Program of Shaanxi Provincial People’s Hospital (2021JY-43).

## Conflict of Interest

The authors declare that the research was conducted in the absence of any commercial or financial relationships that could be construed as a potential conflict of interest.

## Publisher’s Note

All claims expressed in this article are solely those of the authors and do not necessarily represent those of their affiliated organizations, or those of the publisher, the editors and the reviewers. Any product that may be evaluated in this article, or claim that may be made by its manufacturer, is not guaranteed or endorsed by the publisher.

## Abbreviations

ALNM, Axillary lymph node metastasis; SLNM, Sentinel lymph node metastasis; ALND, Axillary lymph node dissection; AUC, Area under the curve; CI, Confidence intervals; DCE, Dynamic contrast-enhanced; MRI, Magnetic resonance imaging; ML, Machine learning; PLR, Positive likelihood ratio; QUADAS-2, Quality Assessment of Diagnostic Accuracy Studies 2; RQS, Radiomics Quality Score; SROC, Summary receiver operating characteristic curve; SLNB, Sentinel lymph node biopsy; NLR, Negative likelihood ratio.
