# Supplementary material for: The Diagnostic Performance of Machine Learning-Based Radiomics of DCE-MRI in Predicting Axillary Lymph Node Metastasis in Breast Cancer: A Meta-Analysis
Source: Front Oncol. 2022 Feb 4;12:799209. doi: 10.3389/fonc.2022.799209 (PMC8854258; doi:10.3389/fonc.2022.799209)

Studies	Sensitivity Estimate (95% C.I.)	TP / (TP + FN)	Specificity Estimate (95% C.I.)	TN / (FP + TN)
Arefan	0.725 (0.617, 0.812)	58/80	0.784 (0.676, 0.863)	58/74
Luo	0.923 (0.828, 0.968)	60/65	0.899 (0.802, 0.951)	62/69
Cui	0.942 (0.836, 0.981)	49/52	0.794 (0.676, 0.876)	50/63
Shan	0.882 (0.736, 0.952)	33/37	0.986 (0.813, 0.999)	35/35
Zhan	0.870 (0.665, 0.957)	20/23	0.714 (0.524, 0.850)	20/28
**Subgroup Siemens (I^2=73.1 % , P=0.005)**	**0.876 (0.765, 0.939)**	**220/257**	**0.821 (0.726, 0.889)**	**225/269**
Chen	0.714 (0.524, 0.850)	20/28	0.923 (0.609, 0.989)	12/13
Han	0.784 (0.651, 0.876)	40/51	0.716 (0.609, 0.803)	58/81
Liu CL	0.833 (0.591, 0.945)	15/18	0.778 (0.615, 0.885)	28/36
Liu	0.688 (0.330, 0.907)	5/7	0.929 (0.423, 0.996)	6/6
Liu	0.632 (0.403, 0.813)	12/19	0.810 (0.588, 0.927)	17/21
Nguyen	0.718 (0.655, 0.774)	158/220	0.471 (0.388, 0.555)	64/136
Ren	0.924 (0.831, 0.968)	61/66	0.793 (0.730, 0.844)	153/193
**Subgroup GE (I^2=53.64 % , P=0.044)**	**0.768 (0.682, 0.837)**	**311/409**	**0.752 (0.606, 0.857)**	**338/486**
**Overall (I^2=67.21 % , P=0.000)**	**0.817 (0.749, 0.869)**	**531/666**	**0.787 (0.693, 0.859)**	**563/755**

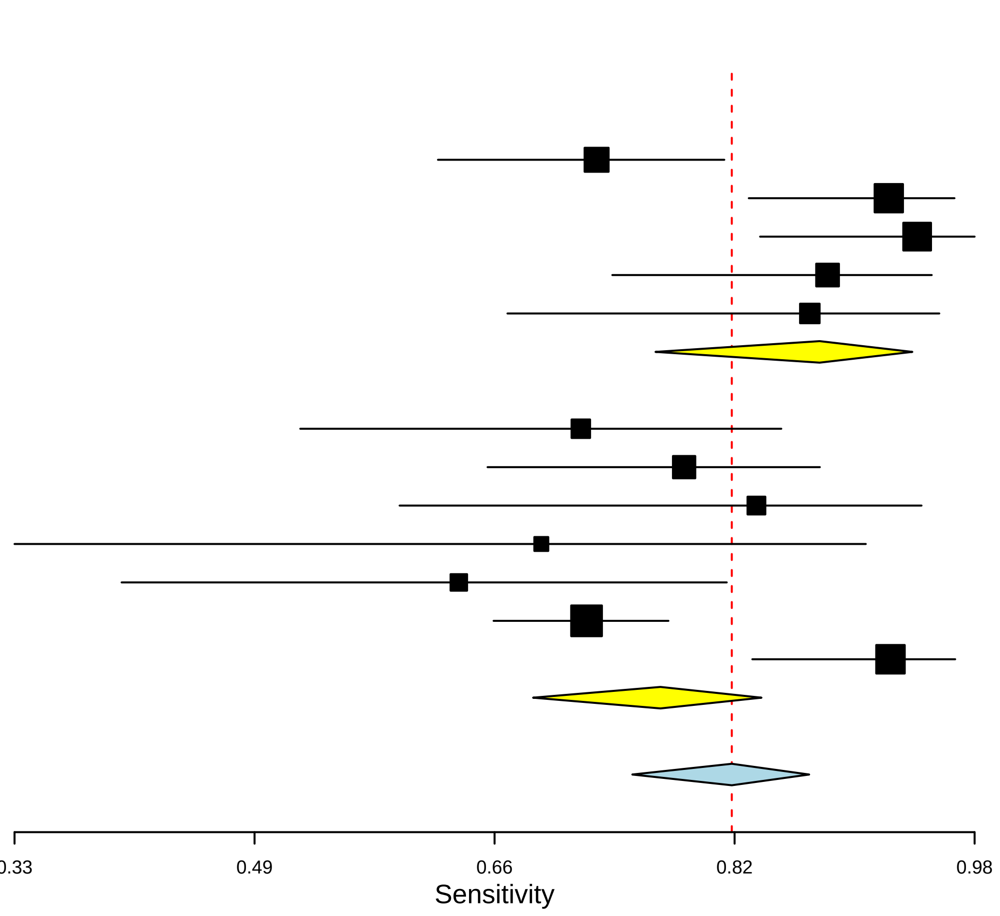

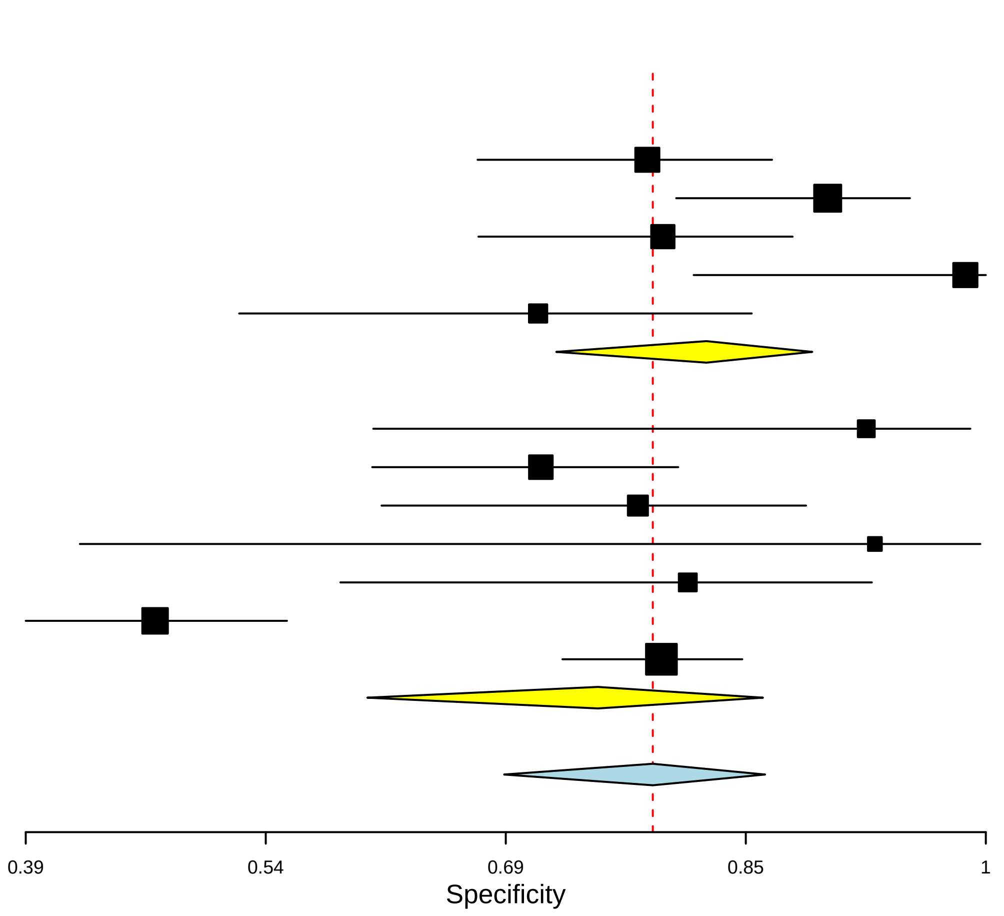

Supplement: Supplementary file 1 [file DataSheet_1.zip › Figure S8-Siemens vs GE.pdf]
