# Supplementary figures and images for: The Diagnostic Performance of Machine Learning-Based Radiomics of DCE-MRI in Predicting Axillary Lymph Node Metastasis in Breast Cancer: A Meta-Analysis
Source: Front Oncol. 2022 Feb 4;12:799209. doi: 10.3389/fonc.2022.799209 (PMC8854258; doi:10.3389/fonc.2022.799209)

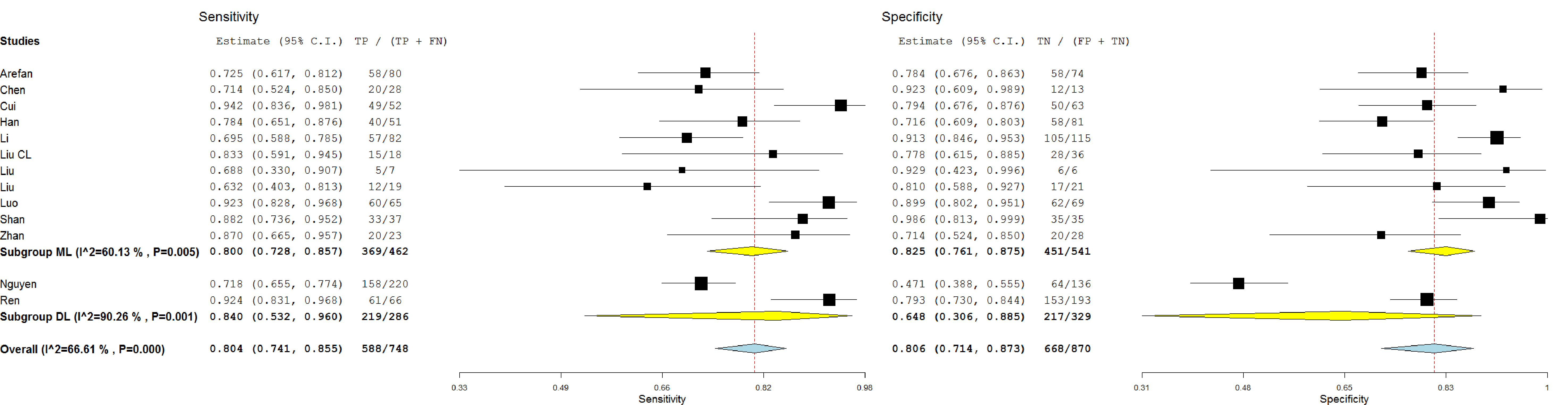

Supplement: Supplementary file 1 [file DataSheet_1.zip › Figure S1-ML vs DL.tif]

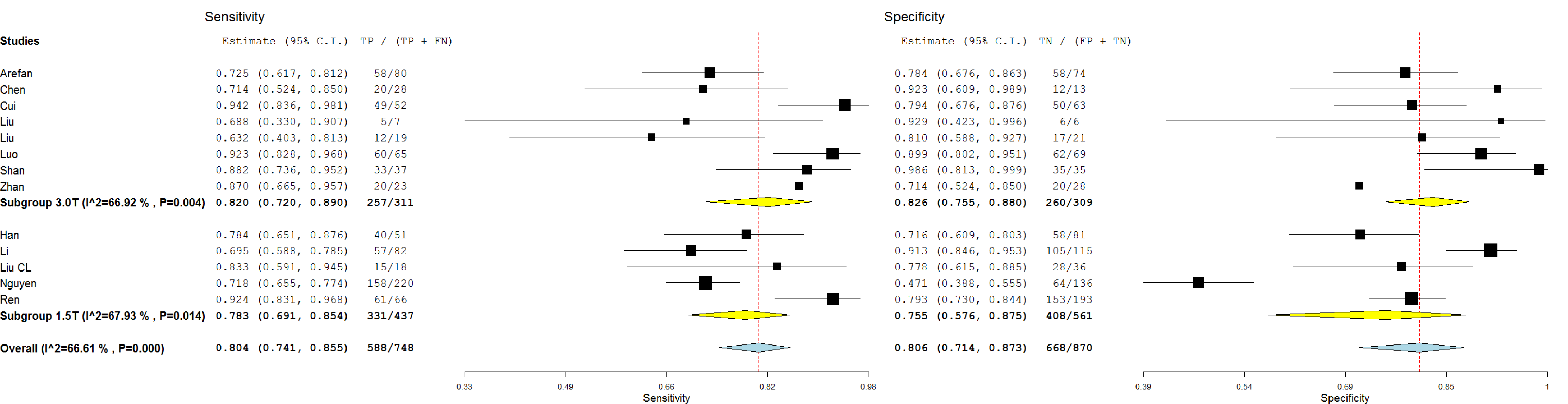

Supplement: Supplementary file 1 [file DataSheet_1.zip › Figure S2-3.0T vs 1.5T.tif]

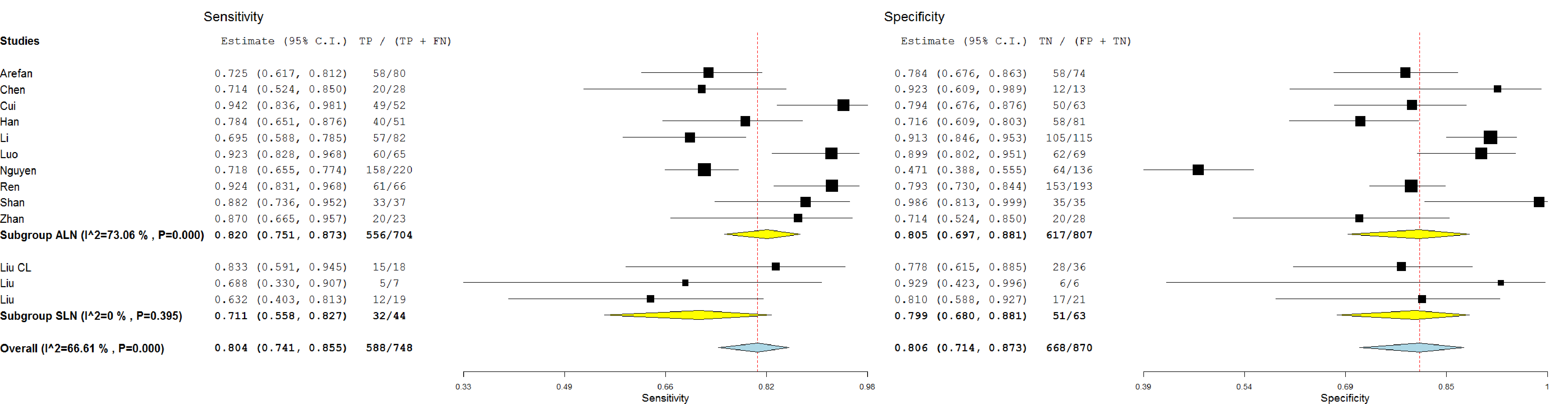

Supplement: Supplementary file 1 [file DataSheet_1.zip › Figure S4-ALN vs SLN.tif]

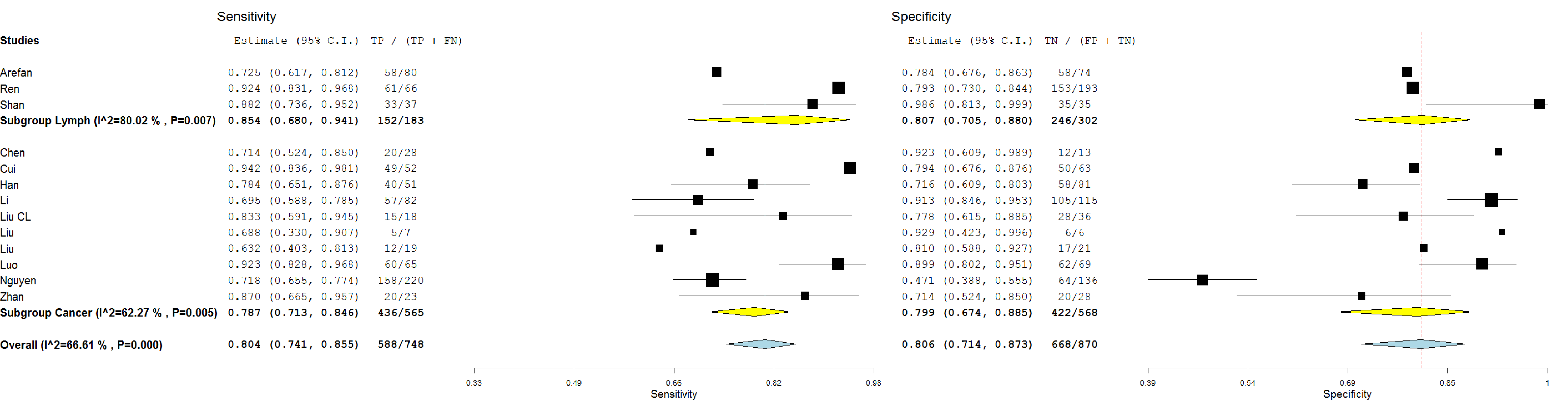

Supplement: Supplementary file 1 [file DataSheet_1.zip › Figure S5-lymph vs cancer.tif]

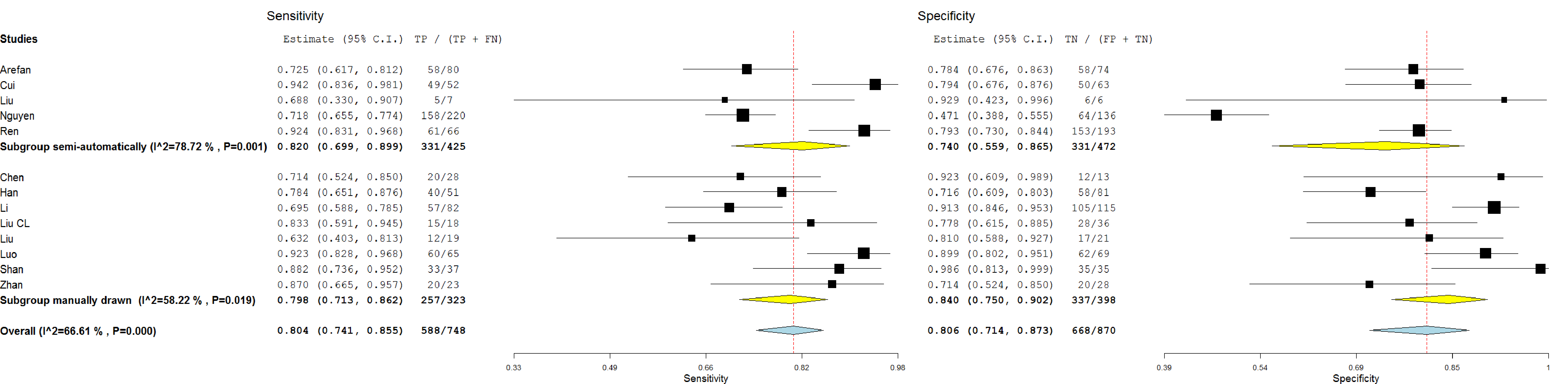

Supplement: Supplementary file 1 [file DataSheet_1.zip › Figure S6-semiautomatic vs manually.tif]

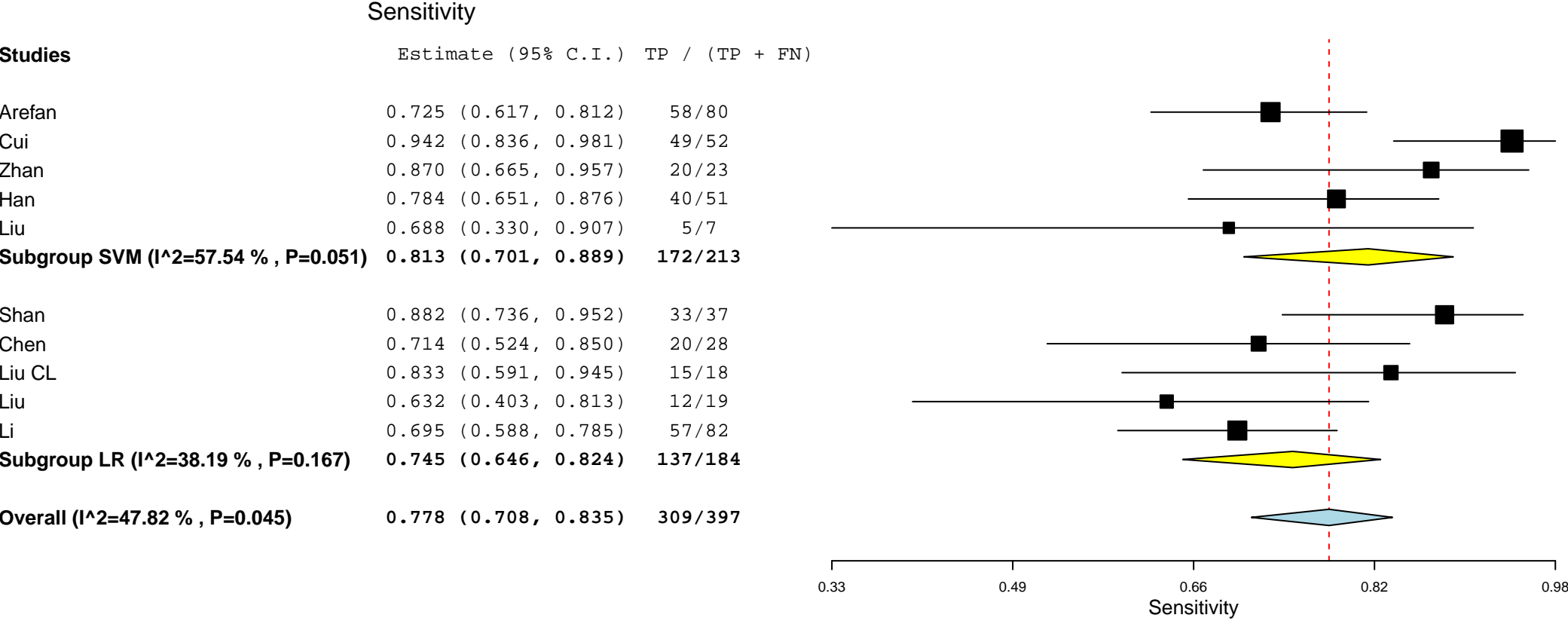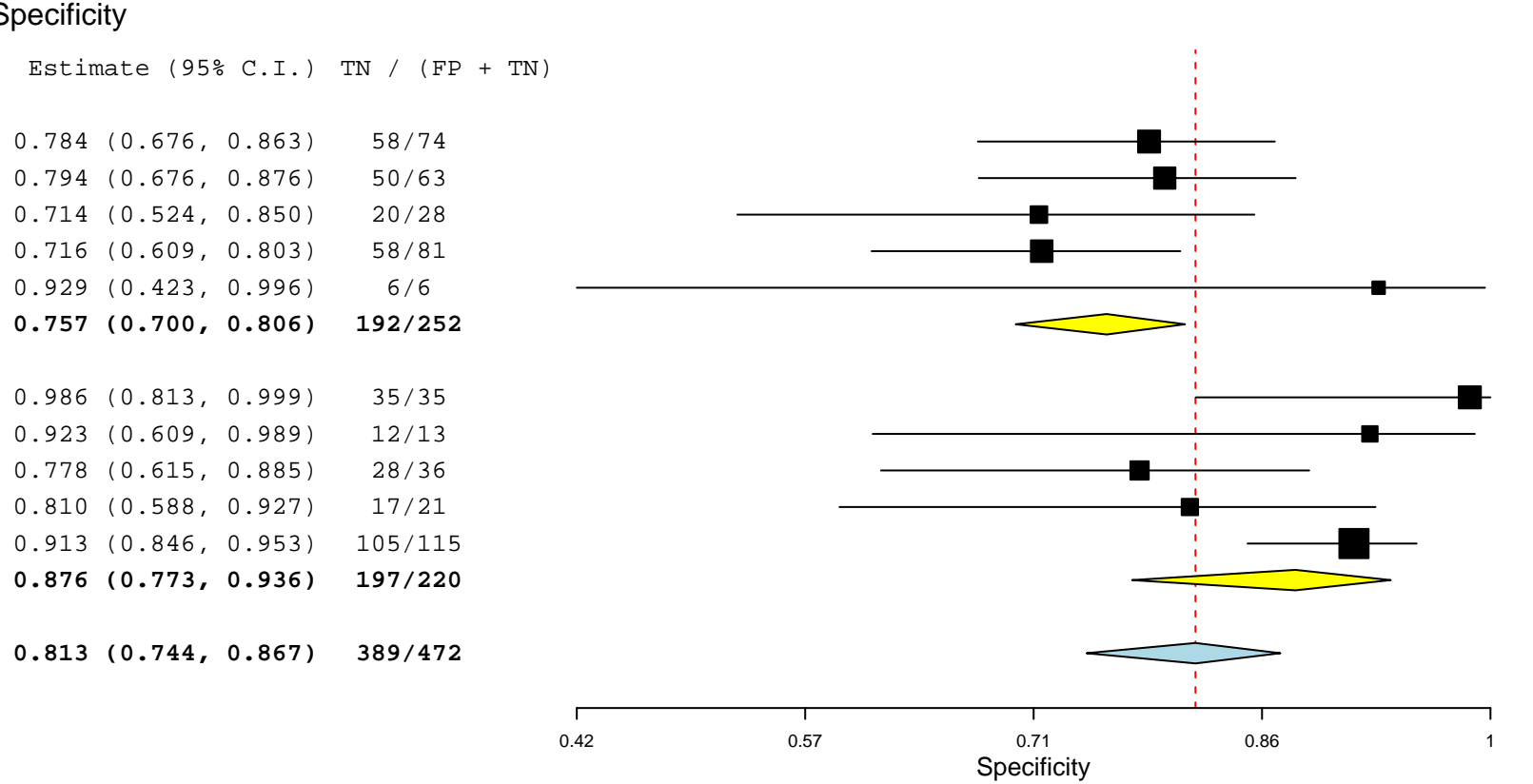

Supplement: Supplementary file 1 [file DataSheet_1.zip › Figure S7-SVM VS LR.pdf]
