# Supplementary material for: The Diagnostic Performance of Machine Learning-Based Radiomics of DCE-MRI in Predicting Axillary Lymph Node Metastasis in Breast Cancer: A Meta-Analysis
Source: Front Oncol. 2022 Feb 4;12:799209. doi: 10.3389/fonc.2022.799209 (PMC8854258; doi:10.3389/fonc.2022.799209)
Supplement: Supplementary file 2 [file Table_1.docx]

**Table S1 The RQS for individual studies and individual ratings for each study**

| **Study** | **Image  Protocol** | **Multiple Segmentation** | | **Phantom Study** | **Multiple Time Points** | | **Feature  Reduction** | | **Non  Radiomics** | **Biological  Correlates** | | **Cut-off** |  |  |
| --- | --- | --- | --- | --- | --- | --- | --- | --- | --- | --- | --- | --- | --- | --- |
| Arefan, 2020 | 1 | 1 | | 1 | 0 | | 3 | | 0 | 0 | | 0 |  |  |
| Chen, 2021 | 1 | 1 | | 1 | 0 | | 3 | | 1 | 0 | | 0 |  |  |
| Cui, 2019 | 1 | 1 | | 0 | 0 | | 3 | | 0 | 0 | | 0 |  |  |
| Han, 2019 | 1 | 0 | | 1 | 0 | | 3 | | 1 | 1 | | 1 |  |  |
| Li, 2021 | 1 | 1 | | 1 | 0 | | 3 | | 1 | 0 | | 0 |  |  |
| Liu CL, 2019 | 1 | 1 | | 0 | 0 | | 3 | | 1 | 0 | | 0 |  |  |
| Liu, 2019 | 1 | 0 | | 0 | 0 | | 3 | | 0 | 0 | | 0 |  |  |
| Liu, 2020 | 1 | 1 | | 0 | 0 | | 3 | | 1 | 0 | | 0 |  |  |
| Luo, 2021 | 1 | 0 | | 1 | 0 | | 3 | | 0 | 0 | | 0 |  |  |
| Nguyen, 2020 | 0 | 1 | | 1 | 1 | | 3 | | 1 | 0 | | 0 |  |  |
| Ren, 2019 | 1 | 0 | | 1 | 0 | | 3 | | 0 | 0 | | 0 |  |  |
| Shan, 2019 | 1 | 1 | | 1 | 0 | | 3 | | 0 | 0 | | 1 |  |  |
| Zhen, 2021 | 1 | 0 | | 0 | 0 | | 3 | | 1 | 0 | | 0 |  |  |
|  |  |  |  |  | |  | |  | | |  | |  |  |
|  |  |  |  |  | |  | |  | | |  | |  |  |
|  |  |  |  |  | |  | |  | | |  | |  |  |
| **Continued** |  |  |  |  | |  | |  | | |  | |  |  |
| **Study** | **Discrimination  and Resampling** | **Prospective** | **Valida-tion** | **Gold Standard** | | **Clinical Utility** | | **Cost- effectiveness** | | | **Open Science** | | **Individual Rating** |  |
| Arefan, 2020 | 0 | 0 | -5 | 2 | | 2 | | 0 | | | 1 | | 16.70% |  |
| Chen, 2021 | 0 | 0 | 2 | 2 | | 2 | | 0 | | | 1 | | 38.80% |  |
| Cui, 2019 | 0 | 0 | -5 | 2 | | 2 | | 0 | | | 1 | | 13.90% |  |
| Han, 2019 | 0 | 0 | 2 | 2 | | 2 | | 0 | | | 1 | | 41.70% |  |
| Li, 2021 | 0 | 0 | 2 | 2 | | 0 | | 0 | | | 1 | | 36.10% |  |
| Liu CL, 2019 | 0 | 0 | 2 | 2 | | 2 | | 0 | | | 1 | | 36.10% |  |
| Liu, 2019 | 0 | 0 | 2 | 2 | | 2 | | 0 | | | 1 | | 30.60% |  |
| Liu, 2020 | 0 | 0 | 2 | 2 | | 2 | | 0 | | | 1 | | 36.10% |  |
| Luo, 2021 | 1 | 0 | -5 | 2 | | 2 | | 0 | | | 1 | | 16.70% |  |
| Nguyen, 2020 | 0 | 0 | 2 | 2 | | 2 | | 1 | | | 1 | | 41.70% |  |
| Ren, 2019 | 0 | 0 | 2 | 2 | | 0 | | 0 | | | 1 | | 27.80% |  |
| Shan, 2019 | 1 | 0 | 2 | 2 | | 2 | | 0 | | | 1 | | 41.70% |  |
| Zhen, 2021 | 0 | 0 | 2 | 2 | | 2 | | 0 | | | 1 | | 33.30% |  |

**Table S2 Detailed two-by-two contingency tables of every study**

| **Study** | **TP** | **FP** | **TN** | **FN** |
| --- | --- | --- | --- | --- |
| Arefan, 2020 | 58 | 16 | 58 | 22 |
| Chen, 2021 | 20 | 1 | 12 | 8 |
| Cui, 2019 | 49 | 13 | 50 | 3 |
| Han, 2019 | 40 | 23 | 58 | 11 |
| Li, 2021 | 57 | 10 | 105 | 25 |
| Liu CL, 2019 | 15 | 8 | 28 | 3 |
| Liu, 2019 | 5 | 0 | 6 | 2 |
| Liu, 2020 | 12 | 4 | 17 | 7 |
| Luo, 2021 | 60 | 7 | 62 | 5 |
| Nguyen, 2020 | 158 | 72 | 64 | 62 |
| Ren, 2019 | 61 | 40 | 153 | 5 |
| Shan, 2019 | 33 | 0 | 35 | 4 |
| Zhan, 2021 | 20 | 8 | 20 | 3 |

TP: true positive; FP: false-positive; FN: false negative; TN: true negative

**Table S3 The results of sensitivity analyses for each study**

| **Eliminated study** | **Sensitivity** | **Specificity** | **PLR** | **NLR** | **DOR** | **AUC** |
| --- | --- | --- | --- | --- | --- | --- |
| Arefan, 2020 | 0.83(0.76,0.88) | 0.83(0.73,0.90) | 4.93(2.99,8.13) | 0.21(0.14,0.30) | 23.96(11.02,52.06) | 0.90(0.87,0.92) |
| Chen, 2021 | 0.83(0.76,0.88) | 0.82(0.73,0.88) | 4.57(2.90,7.20) | 0.21(0.14,0.31) | 21.72(10.24,46.08) | 0.89(0.86,0.92) |
| Cui, 2019 | 0.80(0.74,0.86) | 0.83(0.73,0.90) | 4.76(2.87,7.89) | 0.24(0.17,0.33) | 20.17(9.44,43.07) | 0.88(0.85,0.90) |
| Han, 2019 | 0.82(0.75,0.88) | 0.84(0.74,0.90) | 5.04(3.07,8.27) | 0.21(0.14,0.31) | 23.89(11.04,51.70) | 0.90(0.87,0.92) |
| Li, 2021 | 0.83(0.77,0.88) | 0.81(0.71,0.87) | 4.30(2.75,6.70) | 0.21(0.14,0.30) | 20.82(9.73,44.57) | 0.89(0.86,0.91) |
| Liu CL, 2019 | 0.82(0.75,0.87) | 0.83(0.74,0.90) | 4.86(2.96,8.00) | 0.22(0.15,0.32) | 22.38(10.33,48.51) | 0.89(0.86,0.92) |
| Liu, 2019 | 0.82(0.75,0.88) | 0.82(0.73,0.88) | 4.51(2.91,7.00) | 0.22(0.15,0.32) | 20.82(10.10,42.92) | 0.89(0.86,0.91) |
| Liu, 2020 | 0.83(0.76,0.88) | 0.83(0.73,0.89) | 4.84(2.98,7.85) | 0.21(0.14,0.30) | 23.55(11.03,50.28) | 0.90(0.87,0.92) |
| Luo, 2021 | 0.80(0.74,0.86) | 0.82(0.72,0.89) | 4.43(2.78,7.07) | 0.24(0.17,0.34) | 18.58(9.12,37.86) | 0.88(0.84,0.90) |
| Nguyen, 2020 | 0.83(0.76,0.88) | 0.84(0.78,0.89) | 5.24(3.70,7.41) | 0.20(0.14,0.29) | 25.81(14.39,46.29) | 0.90(0.87,0.93) |
| Ren, 2019 | 0.81(0.74,0.86) | 0.83(0.73,0.90) | 4.80(2.88,7.99) | 0.23(0.17,0.33) | 20.52(9.50,44.32) | 0.88(0.85,0.91) |
| Shan, 2019 | 0.81(0.74,0.87) | 0.80(0.72,0.86) | 4.04(2.80,5.83) | 0.24(0.16,0.34) | 17.17(9.08,32.48) | 0.88(0.84,0.90) |
| Zhan, 2021 | 0.82(0.74,0.87) | 0.83(0.74,0.90) | 4.94(3.01,8.10) | 0.22(0.15,0.32) | 22.49(10.41,48.56) | 0.89(0.86,0.92) |
| Overall | 0.82(0.75,0.87) | 0.83(0.74,0.89) | 4.70(3.01,7.35) | 0.22(0.15,0.31) | 21.56(10.60,43.85) | 0.89(0.86,0.91) |

PLR: positive likelihood ratio; NLR: negative likelihood ratio; DOR: diagnostic odds ratio
